# Retraction: Validation of postnatal growth and retinopathy of prematurity (G-ROP) screening guidelines in a tertiary care hospital of Pakistan: A report from low-middle income country

**DOI:** 10.1371/journal.pone.0327050

**Published:** 2025-06-30

**Authors:** The PLOS One Editors

Following the publication of this article [1,2], PLOS identified concerns regarding the underlying data, and the sample size calculation. Specifically:

- In the underlying data:
  - For multiple infants, identical weight measurements are recorded for the same infant across multiple time points.
  - Some infants appear to show no or minimal weight gain between birth and the later time points, for example with the same weight measurements at birth and 30 days.
  - For all infants, the weight result for day 19 are identical to the weight result on day 20, and separately the weight result on day 29 are identical to the result on day 30.
  - There appear to be differences in the precision of the weight measurements such that many of the weight measurements are reported to 100 grams, several are reported to 10 grams, and a few measurements are reported to 5 grams.
- The Results section of the article states that 123 infants met the G-ROP screening criteria; however, based on the underlying data, 120 infants appeared to meet the criteria.
- During editorial follow-up, the authors provided a copy of the project proposal document. Upon editorial review, it was noted that the sample size calculation differed between the project proposal document and the article, with a required sample size of 291 reported in the project proposal and 148 in the article. Different methods of calculating the sample size, and different populations, prevalence, specificity and sensitivity measurements of ROP were used for the sample size calculation for the project proposal and the article.

Regarding the concerns for the weight data in the underlying data, the corresponding author HT stated that these data were collected from both NICU and outpatient settings. They stated there were different accuracies of the scales used and methods of data recording between the NICU and outpatient units, such that weight was recorded to 1 or 2 decimal places in the NICU and one decimal place in the outpatient clinics, and the scales in each setting rounded the weight measurements differently. They stated that these limitations in weight measurements contributed to the observation that some infants had the same weight measurements at birth and day 30, as well as identical weight measurements for all infants on day 19 and day 20 as well as day 29 and day 30, and that this reflects a limitation of assessing growth trends retrospectively. They also stated that daily weight gains are often minimal due to the slow physiological growth of premature infants. A member of the *PLOS One* Editorial Board reviewed the author’s response regarding the concerns for the weight data, and they stated that the author’s responses did not fully address the lack of weight gain between birth and the later time points for some of the infants. PLOS remains concerned about the reliability and integrity of the weight measurements due to these concerns.

The corresponding author HT stated that the sample size was revised given feasibility constraints and case availability within the study timeframe, and that an interim analysis identified a higher prevalence of ROP in the target population. They stated that the sample size calculation parameters were adjusted to emphasize the sensitivity over specificity in detecting at-risk infants for ROP based on the G-ROP criteria. They stated a post hoc power analysis indicated sufficient power to detect the effect, so further data collection was halted.

In light of the above concerns that question the reliability of the results and conclusions, the *PLOS One* Editors retract this article.

HT, AM, HJ, MAM, SH, and KA agreed with the retraction and apologize for the issues with the published article. RAC and SNAM either did not respond directly or could not be reached.

The Materials and methods section of this article states that the study protocol was reviewed and approved by the Ethical Review Committee of The Aga Khan University Hospital with approval number 2021-6299-18532. During editorial follow-up, the authors provided the ethics review document 2021-6299-18532. This document shows the study was exempt from ethical approval by The Aga Khan University Hospital.
